# Crystal structure of 2,2′′-bis­(2,7-di­chloro-9-hy­droxy-9*H*-fluoren-9-yl)-1,1′:4′,1′′-terphenyl tri­ethyl­amine trisolvate

**DOI:** 10.1107/S2056989015018824

**Published:** 2015-11-04

**Authors:** Henrik Klien, Wilhelm Seichter, Edwin Weber

**Affiliations:** a Institut für Organische Chemie, TU Bergakademie Freiberg, Leipziger Strasse 29, D-09596 Freiberg/Sachsen, Germany

**Keywords:** crystal structure, 2,2′-disubstituted 1,1′:4′,1′′-terphen­yl, tri­ethyl­amine solvate, compound synthesis, hydrogen bonding

## Abstract

In the title solvate, C_44_H_26_Cl_4_O_2_·3C_6_H_15_N, the asymmetric part of the unit cell comprises two halves of the diol mol­ecules, 2,2′′-bis­(2,7-di­chloro-9-hy­droxy-9*H*-fluoren-9-yl)-1,1′:4′,1′′-terphenyl, and three mol­ecules of tri­ethyl­amine, *i. e*. the diol mol­ecules are located on crystallographic symmetry centres. Two of the solvent mol­ecules are disordered over two positions [occupancy ratios of 0.567 (3):0.433 (3) and 0.503 (3):0.497 (3)]. In the diol mol­ecules, the outer rings of the 1,1′:4′,1′′-terphenyl elements are twisted with reference to their central arene ring and the mean planes of the fluorenyl moieties are inclined with respect to the terphenyl ring to which they are connected, the latter making dihedral angles of 82.05 (8) and 82.28 (8)°. The presence of two 9-fluoren-9-ol units attached at positions 2 and 2′′ of the terphenyl moiety induces a ‘folded’ geometry which is stabilized by intra­molecular C—H⋯O hydrogen bonds and π–π stacking inter­actions, the latter formed between the fluorenyl units and the central ring of the terphenyl unit [centroid–centroid distances = 3.559 (1) and 3.562 (1) Å]. The crystal is composed of 1:2 complex units, in which the solvent mol­ecules are associated with the diol mol­ecules *via* O—H⋯N hydrogen bonds, while the remaining solvent mol­ecule is linked to the host by a C—H⋯N hydrogen bond. The given pattern of inter­molecular inter­actions results in formation of chain structures extending along [010].

## Abstract 2

In the crystal structure of 2,2′′-bis­(2,7-di­chloro-9-hy­droxy-9-fluoren­yl)-1,1′:4′,1′′-terphenyl as the tris­(tri­ethyl­amine) solvate, the diol host mol­ecule possesses a ‘folded’ mol­ecular conformation with inversion symmetry. Two of the three solvent mol­ecules form O—H⋯N hydrogen bonds and the third one forms C—H⋯N hydrogen bonds with the host compound.

## Chemical context   

Compounds featuring two bulky 9-hy­droxy-9-fluorenyl moieties laterally attached to a linear central unit such as a biphenyl group (Weber *et al.*, 1993 ▸; Barbour *et al.*, 1993 ▸; Ibragimov *et al.*, 2001 ▸; Skobridis *et al.*, 2007 ▸) or other linear combinations of phenyl­ene and ethyl­ene components (Weber *et al.*, 2002 ▸) are well known for their high ability to form crystalline host–guest inclusions (Weber, 1996 ▸). Both exchange of the central biphenyl axis for a 1,1′:4′,1′′-terphenyl moiety [*cf.* (I)] (Klien *et al.*, 2013 ▸, 2014 ▸) as well as the addition of substituents to the lateral fluorenyl groups in a representative mol­ecule (Bourne *et al.*, 1994 ▸; Caira *et al.*, 1997 ▸; Weber *et al.*, 2002 ▸) have been performed in order to exercise potential control of the mol­ecular packing in the crystal and thus on the inclusion behavior towards selected guests. Along these lines, aside from conventional hydrogen bonding (Braga & Grepioni, 2004 ▸), Cl⋯Cl supra­molecular inter­actions (Awwadi *et al.*, 2006 ▸) have recently been found to support crystal engineering of an intended lattice structure (Metrangolo *et al.*, 2008 ▸; Mukherjee *et al.*, 2014 ▸). Being associated with this, a corresponding structural modification of the parent mol­ecule (I) by chloro substitution, giving rise to compound (II), presented a promising study. Hence, the synthesis of (II) was undertaken and is reported on here in detail. We were also successful in preparing a crystalline inclusion solvate of (II) with tri­ethyl­amine, the title compound (II*a*), the crystal structure of which is described and discussed and compared to the structures of related compounds.
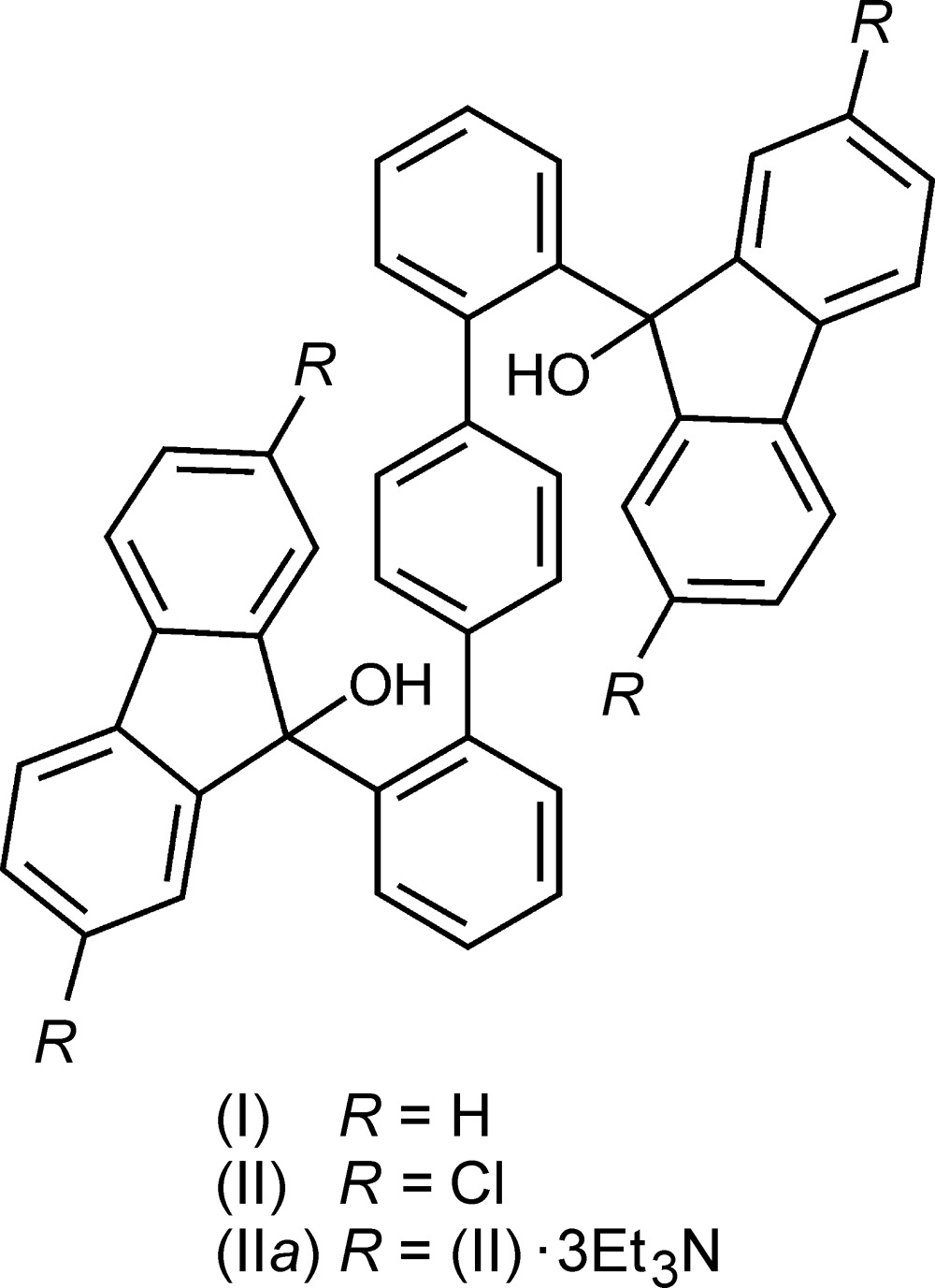


## Structural commentary   

**Figure 1:**
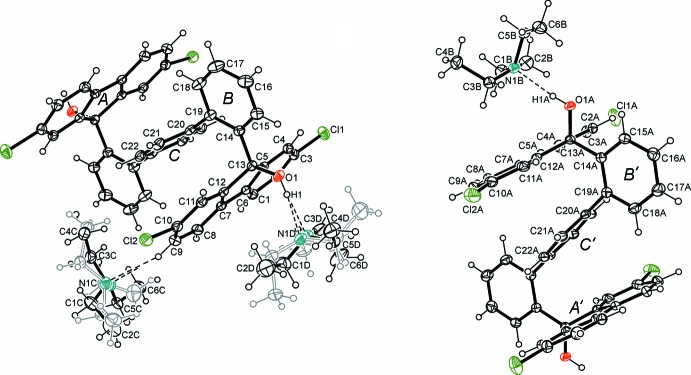
A perspective view of the title solvate (II*a*) including the atom numbering and ring specification. Anisotropic displacement parameters for non-hydrogen atoms are drawn at the 50% probability level. Dashed lines represent hydrogen-bonding inter­actions. The mol­ecules occupy the symmetry centers *x* + 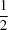, *y*, *z* and *x* + 1, *y* + 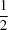, *z* + 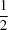)

**Figure 2:**
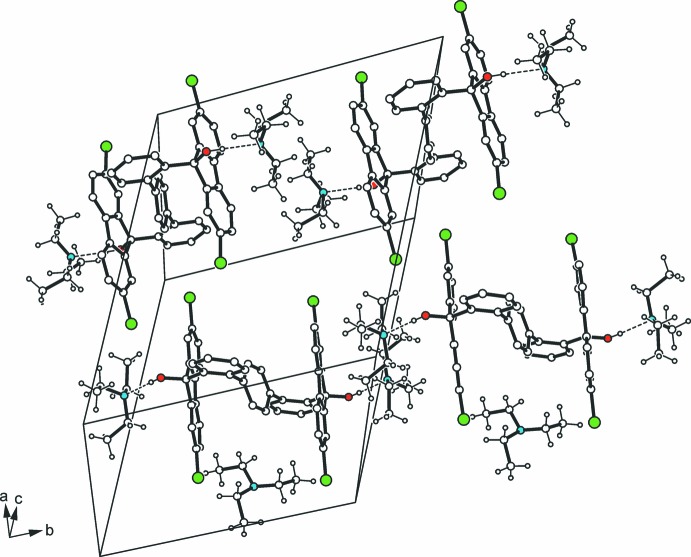
The packing of the title compound (II*a*) in the unit cell. Hydrogen bonds are shown as dashed lines.

The title solvate (II*a*) crystallizes in the space group *P* 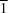 with two halves of the diol mol­ecules (centred at *x* + 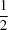, *y*, *z* and *x* + 1, *y* + 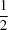, *z* + 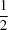) and three mol­ecules of tri­ethyl­amine in the asymmetric unit, *i.e.* the diol mol­ecules occupy crystallographic inversion centres (Fig. 1 ▸). Two of the solvent mol­ecules are disordered over two positions with occupancy ratios of 0.567 (3):0.433 (3) and 0.503 (3):0.497 (3). A perspective view of the mol­ecular structure including ring specification is depicted in Fig. 1 ▸. The fluorenyl moieties of the diol mol­ecules show a slight distortion from strict planarity with the largest distances from the best plane being 0.027 (1) and −0.030 (1) Å for C7 and C10, respectively, and 0.059 (1) and −0.068 (1) Å for C8*A* and C11*A*. respectively The mol­ecules adopt a ‘folded’ geometry which is stabilized by two types of inter­molecular inter­actions. The OH oxygen atoms form relatively strong C—H⋯O hydrogen bonds [*d*(H⋯O) 2.22, 2.23 Å] (Desiraju & Steiner, 1999 ▸) which enforce a nearly orthogonal orientation of the fluorenyl moieties with respect to the terphenyl ring, to which they are attached: the dihedral angles between the five-membered ring of the fluorenyl unit defined by C1–C13 (or C1*A*–C13*A*) and the six-membered rings of the terphenyl unit defined by C14–C19 (or C14*A*–C19*A*) are 82.05 (8) or 82.28 (8)°, respectively. Moreover, the location of the central ring of the terphenyl unit between the fluorenyl units [ring centroid distances = 3.559 (1) and 3.562 (1) Å] indicate the presence of π–π stacking inter­actions (James, 2004 ▸; Martinez & Iverson, 2012 ▸) between these mol­ecular parts. These cooperative intra­molecular inter­actions enforce a nearly orthogonal arrangement of the outer ring (*B* or *B*′) with respect to the inner ring (*A* or *A*′) (Fig. 2 ▸) of the terphenyl unit [inter-ring dihedral angles = 76.3 (1) and 79.3 (1)°, respectively].

## Supra­molecular features   

According to the distinct acceptor character of the solvent species, the crystal structure is constructed of 1:2 complex units with the nitro­gen atom of the solvent hydrogen-bonded to the OH hydrogen atom of the diol host [*d*(H⋯N) 1.91–1.95 Å] (Table 1 ▸). The remaining solvent mol­ecule is connected to the host *via* C—H⋯O hydrogen bonding [*d*(H⋯N) 2.54; 2.60 Å], giving an overall chain structure extending along [010] (Fig. 2 ▸). Inter­actions involving the chlorine atoms are not perceptible. A comparative consideration regarding the geometric features of the diol mol­ecule in the present structure and the solvent-free structure of the corresponding unsubstituted compound (I) and its derivatives bearing alkyl groups in the 2- and 7-positions of the fluorenyl moieties as well as a variety of their inclusion structures (Klien *et al.* 2013 ▸, 2014 ▸), reveals restricted conformational flexibility. This means that neither the presence of substituents nor the nature of the included solvent species markedly affect the conformation of the diol mol­ecule. Obviously, the mol­ecular geometries in the solid-state structures follow close-packing requirements and, to a lesser extent, association effects.

## Database survey   

A search of the Cambridge Structural Database (Groom & Allen, 2014 ▸) for the 2,2′′-disubstituted *p*-terphenyls yielded eleven hits, namely 4,4′′′’-bis­(4-meth­oxy­benzo­yl)-1,1′:2′,1′′:4′′,1′′′:2′′′,1′′′’-quinquephenyl (Debroy *et al.*, 2009 ▸), 2,2′′-bis­(bromo­meth­yl)-*p*-terphenyl (Jones & Kuś, 2005 ▸), 2,2′′-dimethyl-*p*-terphenyl (Lunazzi *et al.*, 2005 ▸), 2′,4′′,2′′′-quinquephenyl (Toussaint, 1966 ▸), 9,9′-(1,1′:4′,1′′-terphenyl-2,2′′-di­yl)bis­(9*H*-fluorene-9-ol) bis­(di­ethyl­amine) clathrate (Klien *et al.*, 2013 ▸), 9,9′-(1,1′:4′,1′′-terphenyl-2,2′′-di­yl)bis­(9*H*-fluor­ene-9-ol) bis­(propan-1-ol) clathrate (Klien *et al.*, 2013 ▸), 9,9′-(1,1′:4′,1′′-terphenyl-2,2′′-di­yl)bis­(9*H*-fluorene-9-ol) bis­(but­an-1-ol) clathrate (Klien *et al.*, 2013 ▸), 9,9′-(1,1′:4′,1′′-terphenyl-2,2′′-di­yl)bis­(9*H*-fluorene-9-ol) bis­(ethanol) clathrate (Klien *et al.*, 2013 ▸), 9,9′-(1,1′:4′,1′′-terphenyl-2,2′′-di­yl)bis­(2,7-di-*t*-butyl-9*H*-fluorene-9-ol) bis­(propan-1-ol) clathrate (Klien *et al.*, 2013 ▸), 9,9′-(1,1′:4′,1′′-terphenyl-2,2′′-di­yl)bis­(2,7-di-*t*-butyl-9*H*-fluorene-9-ol) bis­(di­ethyl­amine) clathrate (Klien *et al.*, 2013 ▸), 9,9′-(1,1′:4′,1′′-terphenyl-2,2′′-di­yl)bis­(2,7-di-*t*-butyl-9*H*-fluorene-9-ol) bis­(butan-1-ol) clathrate (Klien *et al.*, 2013 ▸). In all cases, the terphenyl framework adopts a twisted conformation, which in the case of the bis­fluorenyl-substituted derivatives is stabilized by intra­molecular π–π arene stacking and C—H⋯O hydrogen bonds. The crystal structures of the clathrates, which involve protic guest species in general, are constructed of 1:2 host–guest complexes with the complex components associated with other *via* O—H⋯O and O—H⋯N hydrogen bonds. Both of these features, regarding mol­ecular conformation and supra­molecular inter­actions, reappear in the title compound.

## Synthesis and crystallization   

The unsolvated compound (II) was prepared by addition of a solution of *n-*butyl­lithium (1.6 *M* in hexane, 1.5 ml, 2.3 mmol) to a cold solution (195 K) of 2,2′′-di­iodo-1,1′:4′,1′′-terphenyl (0.5 g, 1.0 mmol) in 20 ml of dry THF. After 45 min of stirring, 4,4′-di­chloro­benzo­phenone (0.52 g, 2.1 mmol), dissolved in 4 ml benzene and 15 ml THF, was added. The colourless reaction mixture was warmed to room temperature and stirred for 4 h. The solution was extracted twice with diethyl ether. The combined organic extracts were washed with water, dried over anhydrous sodium sulfate and concentrated under reduced pressure. Colourless crystals were isolated by recrystallization from hexane (yield: 7.0%). M.p. 543–546 K; ESI–MS [*M* + H]^−^ *m*/*z* 731.3. IR (KBr) *ν* (cm^−1^) 3547, 3056, 3025, 1913, 1641, 1591, 1575, 1489, 1331, 1182, 1157, 1097, 1014, 919, 903, 840, 761. ^1^H NMR (500.1 MHz; CDCl_3_): δ = 2.84 (2H, *s*, OH, 6.67 (4H, *s*, ArH), 6.75 (2H, *d*, ^3^ *J*_HH_ = 7.80 Hz, ArH), 7.09 (8H, *d*, ^3^ *J*_HH_ = 8.50 Hz, ArH), 7.11 (2H, *d*, ^3^ *J*_HH_ = 8.00 Hz, ArH), 7.22 (2H, *t*, ^3^ *J*_HH_ = 7.50 Hz, ArH), 7.26 (8H, *d*, ^3^ *J*_HH_ = 9.00 Hz, ArH), 7.32 (2H, *t*, ^3^ *J*_HH_ = 7.25 Hz, ArH). ^13^C NMR (125.7 MHz, CDCl_3_): δ = 82.68 (C-OH), 126.89, 127.43, 128.10, 129.11, 129.33, 129.83, 133.40, 140.24, 141.01, 144.06, 145.58 (Ar-C). EA calculated for C_44_H_30_O_2_Cl_4_: C 72.1, H 4.1%; found: C 72.2, H 4.4%. Crystals of (II*a*) suitable for X-ray diffraction were obtained from a solution of (II) in tri­ethyl­amine upon slow evaporation of the solvent at room temperature.

## Refinement details   

Crystal data, data collection and structure refinement details are summarized in Table 2 ▸. All H atoms were placed geometrically in idealized positions and allowed to ride on their parent atoms, with C—H = 0.95 and 0.98 Å and *U*_iso_(H) = 1.2*U*_eq_(C) for aromatic and methyl­ene, with C—H = 0.98 and O—H = 0.84 Å and *U*_iso_(H) = 1.5*U*_eq_(C) for methyl and hy­droxy groups, respectively. Two mol­ecules of tri­ethyl­amine are each disordered over two positions with occupancy ratios of 0.567 (3):0.433 (3) and 0.503 (3):0.497 (3). They were modelled with restrained bond lengths based on average values of 1.47 (1) Å for N—C and 1.53 (1) Å for C—C bonds.

## Supplementary Material

Crystal structure: contains datablock(s) I, New_Global_Publ_Block. DOI: 10.1107/S2056989015018824/zs2345sup1.cif

Structure factors: contains datablock(s) I. DOI: 10.1107/S2056989015018824/zs2345Isup2.hkl

Supporting information file. DOI: 10.1107/S2056989015018824/zs2345Isup3.cml

CCDC reference: 1430018

Additional supporting information:  crystallographic information; 3D view; checkCIF report

## Figures and Tables

**Table 1:** Hydrogen-bond geometry (Å, °)

*D*—H⋯*A*	*D*—H	H⋯*A*	*D*⋯*A*	*D*—H⋯*A*
O1—H1⋯N1*D* ^i^	0.84	1.95	2.781 (2)	171
O1—H1⋯N1*DA* ^i^	0.84	1.91	2.731 (2)	164
O1*A*—H1*A*⋯N1*B* ^ii^	0.84	1.94	2.766 (2)	167
C4—H4⋯O1*A* ^iii^	0.95	2.54	3.489 (2)	175
C4*A*—H4*A*⋯O1^iv^	0.95	2.47	3.403 (2)	168
C9—H9⋯N1*C* ^v^	0.95	2.54	3.459 (2)	163
C9—H9⋯N1*CA* ^v^	0.95	2.60	3.519 (2)	162

Symmetry codes: (i) 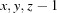; (ii) 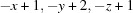; (iii) 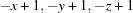; (iv) 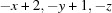; (v) 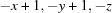.

**Table 2:** Experimental details

Crystal data	
Chemical formula	C_44_H_26_Cl_4_O_2_·3C_6_H_15_N
*M*_r_	1032.01
Crystal system, space group	Triclinic, *P* 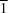
Temperature (K)	100
*a*, *b*, *c* (Å)	14.5995 (2), 14.8094 (2), 15.7705 (3)
α, β, γ (°)	68.373 (1), 66.837 (1), 67.558 (1)
*V* (Å^3^)	2800.13 (8)
*Z*	2
Radiation type	Mo *K*α
μ (mm^−1^)	0.26
Crystal size (mm)	0.42 × 0.40 × 0.23

Data collection	
Diffractometer	Bruker CCD area detector
Absorption correction	Multi-scan (*SADABS*; Bruker, 2008 ▸)
*T*_min_, *T*_max_	0.900, 0.943
No. of measured, independent and observed [*I* > 2σ(*I*)] reflections	53831, 14031, 11477
*R*_int_	0.025
(sin θ/λ)_max_ (Å^−1^)	0.669

Refinement	
*R*[*F* ^2^ > 2σ(*F* ^2^)], *wR*(*F* ^2^), *S*	0.040, 0.113, 1.01
No. of reflections	14031
No. of parameters	773
No. of restraints	24
H-atom treatment	H-atom parameters constrained
Δρ_max_, Δρ_min_ (e Å^−3^)	0.59, −0.59

Computer programs: *APEX2* and *SAINT* (Bruker, 2008 ▸), *SHELXS97* and *SHELXTL* (Sheldrick, 2008 ▸), *SHELXL2013* (Sheldrick, 2015 ▸) and *ORTEP-3 for Windows* (Farrugia, 2012 ▸).

## supplementary crystallographic information

### Crystal data

**Table:**

C_44_H_26_Cl_4_O_2_·3C_6_H_15_N	*Z* = 2
*M**_r_* = 1032.01	*F*(000) = 1096
Triclinic, *P*1	*D*_x_ = 1.224 Mg m^−^^3^
*a* = 14.5995 (2) Å	Mo *K*α radiation, λ = 0.71073 Å
*b* = 14.8094 (2) Å	Cell parameters from 9853 reflections
*c* = 15.7705 (3) Å	θ = 2.4–31.3°
α = 68.373 (1)°	µ = 0.26 mm^−^^1^
β = 66.837 (1)°	*T* = 100 K
γ = 67.558 (1)°	Plate, colourless
*V* = 2800.13 (8) Å^3^	0.42 × 0.40 × 0.23 mm

### Data collection

**Table:**

Bruker CCD area detector diffractometer	11477 reflections with *I* > 2σ(*I*)
φ and ω scans	*R*_int_ = 0.025
Absorption correction: multi-scan (*SADABS*; Bruker, 2008)	θ_max_ = 28.4°, θ_min_ = 1.5°
*T*_min_ = 0.900, *T*_max_ = 0.943	*h* = −19→19
53831 measured reflections	*k* = −19→19
14031 independent reflections	*l* = −21→21

### Refinement

**Table:**

Refinement on *F*^2^	24 restraints
Least-squares matrix: full	Hydrogen site location: inferred from neighbouring sites
*R*[*F*^2^ > 2σ(*F*^2^)] = 0.040	H-atom parameters constrained
*wR*(*F*^2^) = 0.113	*w* = 1/[σ^2^(*F*_o_^2^) + (0.0538*P*)^2^ + 1.6165*P*] where *P* = (*F*_o_^2^ + 2*F*_c_^2^)/3
*S* = 1.01	(Δ/σ)_max_ = 0.001
14031 reflections	Δρ_max_ = 0.59 e Å^−^^3^
773 parameters	Δρ_min_ = −0.59 e Å^−^^3^

### Special details

**Table:**

Geometry. All e.s.d.'s (except the e.s.d. in the dihedral angle between two l.s. planes) are estimated using the full covariance matrix. The cell e.s.d.'s are taken into account individually in the estimation of e.s.d.'s in distances, angles and torsion angles; correlations between e.s.d.'s in cell parameters are only used when they are defined by crystal symmetry. An approximate (isotropic) treatment of cell e.s.d.'s is used for estimating e.s.d.'s involving l.s. planes.

### Fractional atomic coordinates and isotropic or equivalent isotropic displacement parameters (Å^2^)

**Table:**

	*x*	*y*	*z*	*U*_iso_*/*U*_eq_	Occ. (<1)
Cl1	0.60638 (3)	0.02641 (3)	0.36155 (3)	0.02782 (9)	
Cl2	0.59812 (3)	0.31311 (3)	−0.33671 (3)	0.02962 (9)	
O1	0.78230 (7)	0.18376 (8)	−0.03539 (7)	0.0199 (2)	
H1	0.7514	0.2398	−0.0220	0.030*	
C1	0.63440 (10)	0.12716 (10)	0.08381 (9)	0.0163 (2)	
C2	0.65868 (10)	0.07862 (10)	0.16874 (10)	0.0188 (3)	
H2	0.7279	0.0419	0.1697	0.023*	
C3	0.57796 (11)	0.08549 (11)	0.25318 (10)	0.0199 (3)	
C4	0.47565 (10)	0.13766 (11)	0.25341 (10)	0.0207 (3)	
H4	0.4220	0.1398	0.3120	0.025*	
C5	0.45285 (10)	0.18656 (11)	0.16705 (10)	0.0198 (3)	
H5	0.3835	0.2226	0.1661	0.024*	
C6	0.53259 (10)	0.18211 (10)	0.08225 (10)	0.0171 (3)	
C7	0.53127 (10)	0.22501 (10)	−0.01767 (10)	0.0177 (3)	
C8	0.45058 (11)	0.28265 (11)	−0.05765 (11)	0.0225 (3)	
H8	0.3818	0.3026	−0.0180	0.027*	
C9	0.47169 (12)	0.31085 (11)	−0.15657 (11)	0.0250 (3)	
H9	0.4175	0.3500	−0.1851	0.030*	
C10	0.57288 (12)	0.28102 (11)	−0.21279 (10)	0.0221 (3)	
C11	0.65509 (11)	0.22404 (10)	−0.17454 (10)	0.0196 (3)	
H11	0.7240	0.2051	−0.2144	0.024*	
C12	0.63267 (10)	0.19620 (10)	−0.07658 (10)	0.0172 (3)	
C13	0.70829 (10)	0.13336 (10)	−0.01747 (9)	0.0163 (2)	
C14	0.76933 (10)	0.03037 (10)	−0.04016 (9)	0.0180 (3)	
C15	0.87711 (11)	0.00223 (11)	−0.06259 (10)	0.0220 (3)	
H15	0.9098	0.0451	−0.0596	0.026*	
C16	0.93772 (12)	−0.08693 (12)	−0.08910 (11)	0.0288 (3)	
H16	1.0111	−0.1043	−0.1046	0.035*	
C17	0.89114 (13)	−0.15025 (13)	−0.09285 (13)	0.0339 (4)	
H17	0.9321	−0.2113	−0.1111	0.041*	
C18	0.78417 (13)	−0.12403 (12)	−0.06975 (12)	0.0297 (3)	
H18	0.7526	−0.1680	−0.0724	0.036*	
C19	0.72127 (11)	−0.03479 (11)	−0.04260 (10)	0.0211 (3)	
C20	0.60696 (11)	−0.01430 (10)	−0.02017 (10)	0.0201 (3)	
C21	0.54278 (11)	−0.03021 (11)	0.07381 (10)	0.0212 (3)	
H21	0.5716	−0.0514	0.1249	0.025*	
C22	0.43748 (11)	−0.01550 (11)	0.09362 (10)	0.0216 (3)	
H22	0.3950	−0.0258	0.1580	0.026*	
Cl1A	0.92106 (3)	0.86051 (4)	0.13781 (3)	0.03593 (11)	
Cl2A	0.87208 (4)	0.64484 (3)	0.84734 (3)	0.04199 (12)	
O1A	0.71671 (7)	0.87036 (7)	0.52729 (7)	0.01877 (19)	
H1A	0.7474	0.9119	0.5195	0.028*	
C1A	0.87109 (10)	0.78873 (10)	0.41745 (10)	0.0163 (2)	
C2A	0.85448 (10)	0.81792 (10)	0.32984 (10)	0.0186 (3)	
H2A	0.7870	0.8343	0.3246	0.022*	
C3A	0.94014 (11)	0.82247 (11)	0.24930 (10)	0.0219 (3)	
C4A	1.04014 (11)	0.79780 (11)	0.25515 (11)	0.0237 (3)	
H4A	1.0974	0.7998	0.1989	0.028*	
C5A	1.05506 (10)	0.77022 (11)	0.34418 (11)	0.0213 (3)	
H5A	1.1225	0.7541	0.3494	0.026*	
C6A	0.97026 (10)	0.76652 (10)	0.42527 (10)	0.0178 (3)	
C7A	0.96262 (11)	0.73781 (10)	0.52698 (10)	0.0192 (3)	
C8A	1.03705 (12)	0.71089 (11)	0.57298 (12)	0.0265 (3)	
H8A	1.1068	0.7106	0.5373	0.032*	
C9A	1.00714 (13)	0.68452 (12)	0.67219 (12)	0.0308 (4)	
H9A	1.0565	0.6669	0.7049	0.037*	
C10A	0.90563 (13)	0.68397 (12)	0.72328 (11)	0.0284 (3)	
C11A	0.82964 (12)	0.71172 (11)	0.67874 (11)	0.0239 (3)	
H11A	0.7600	0.7116	0.7147	0.029*	
C12A	0.85985 (11)	0.73933 (10)	0.58041 (10)	0.0184 (3)	
C13A	0.79079 (10)	0.77627 (10)	0.51543 (9)	0.0162 (2)	
C14A	0.73042 (10)	0.70199 (10)	0.53606 (9)	0.0166 (2)	
C15A	0.62299 (10)	0.73819 (11)	0.55279 (10)	0.0199 (3)	
H15A	0.5905	0.8085	0.5461	0.024*	
C16A	0.56284 (11)	0.67338 (12)	0.57902 (11)	0.0245 (3)	
H16A	0.4899	0.6995	0.5902	0.029*	
C17A	0.60905 (11)	0.57107 (12)	0.58886 (11)	0.0269 (3)	
H17A	0.5681	0.5262	0.6082	0.032*	
C18A	0.71616 (11)	0.53410 (11)	0.57025 (11)	0.0238 (3)	
H18A	0.7478	0.4637	0.5765	0.029*	
C19A	0.77833 (10)	0.59803 (10)	0.54256 (9)	0.0175 (3)	
C20A	0.89299 (10)	0.55012 (10)	0.52056 (10)	0.0167 (2)	
C21A	0.95553 (10)	0.55419 (10)	0.42644 (10)	0.0186 (3)	
H21A	0.9256	0.5909	0.3756	0.022*	
C22A	1.06098 (10)	0.50521 (10)	0.40625 (10)	0.0186 (3)	
H22A	1.1023	0.5094	0.3417	0.022*	
N1B	0.21028 (9)	0.98034 (9)	0.47727 (9)	0.0206 (2)	
C1B	0.11268 (11)	0.96584 (13)	0.55043 (11)	0.0272 (3)	
H1B1	0.0968	0.9098	0.5440	0.033*	
H1B2	0.0559	1.0279	0.5385	0.033*	
C2B	0.11490 (14)	0.94238 (14)	0.65192 (12)	0.0339 (4)	
H2B1	0.1588	0.8735	0.6697	0.051*	
H2B2	0.0443	0.9479	0.6955	0.051*	
H2B3	0.1430	0.9905	0.6561	0.051*	
C3B	0.19718 (12)	1.02007 (11)	0.38051 (11)	0.0238 (3)	
H3B1	0.2584	1.0438	0.3357	0.029*	
H3B2	0.1358	1.0798	0.3810	0.029*	
C4B	0.18392 (13)	0.94741 (13)	0.34101 (12)	0.0294 (3)	
H4B1	0.2465	0.8903	0.3347	0.044*	
H4B2	0.1726	0.9829	0.2783	0.044*	
H4B3	0.1241	0.9224	0.3847	0.044*	
C5B	0.29651 (11)	0.88733 (11)	0.48674 (11)	0.0239 (3)	
H5B1	0.2804	0.8329	0.4777	0.029*	
H5B2	0.3021	0.8660	0.5523	0.029*	
C6B	0.40009 (12)	0.89960 (13)	0.41604 (12)	0.0298 (3)	
H6B1	0.3980	0.9125	0.3512	0.045*	
H6B2	0.4549	0.8376	0.4300	0.045*	
H6B3	0.4144	0.9566	0.4212	0.045*	
N1C	0.7405 (9)	0.5936 (6)	0.2422 (6)	0.0241 (7)	0.433 (3)
C1C	0.7208 (4)	0.5371 (4)	0.3433 (3)	0.0351 (10)	0.433 (3)
H1C1	0.6697	0.5835	0.3830	0.042*	0.433 (3)
H1C2	0.7860	0.5122	0.3607	0.042*	0.433 (3)
C2C	0.6805 (5)	0.4481 (6)	0.3656 (7)	0.0465 (19)	0.433 (3)
H2C1	0.6252	0.4693	0.3362	0.070*	0.433 (3)
H2C2	0.6531	0.4236	0.4349	0.070*	0.433 (3)
H2C3	0.7372	0.3937	0.3403	0.070*	0.433 (3)
C3C	0.7547 (3)	0.6915 (3)	0.2294 (3)	0.0287 (9)	0.433 (3)
H3C1	0.7755	0.7248	0.1603	0.034*	0.433 (3)
H3C2	0.8118	0.6794	0.2547	0.034*	0.433 (3)
C4C	0.6581 (11)	0.7621 (9)	0.2788 (9)	0.032 (2)	0.433 (3)
H4C1	0.5981	0.7635	0.2644	0.048*	0.433 (3)
H4C2	0.6667	0.8304	0.2560	0.048*	0.433 (3)
H4C3	0.6472	0.7382	0.3479	0.048*	0.433 (3)
C5C	0.8334 (3)	0.5338 (3)	0.1848 (3)	0.0249 (9)	0.433 (3)
H5C1	0.8277	0.4643	0.2023	0.030*	0.433 (3)
H5C2	0.8950	0.5294	0.1999	0.030*	0.433 (3)
C6C	0.8489 (12)	0.5789 (8)	0.0782 (6)	0.0329 (16)	0.433 (3)
H6C1	0.7852	0.5905	0.0638	0.049*	0.433 (3)
H6C2	0.9061	0.5319	0.0433	0.049*	0.433 (3)
H6C3	0.8653	0.6432	0.0587	0.049*	0.433 (3)
N1CA	0.7332 (7)	0.5984 (5)	0.2574 (5)	0.0241 (7)	0.567 (3)
C1CA	0.7446 (3)	0.4958 (3)	0.3206 (3)	0.0325 (8)	0.567 (3)
H1C3	0.7813	0.4879	0.3652	0.039*	0.567 (3)
H1C4	0.7883	0.4467	0.2816	0.039*	0.567 (3)
C2CA	0.6425 (4)	0.4705 (4)	0.3782 (5)	0.0396 (11)	0.567 (3)
H2C4	0.5999	0.5168	0.4192	0.059*	0.567 (3)
H2C5	0.6558	0.4008	0.4178	0.059*	0.567 (3)
H2C6	0.6059	0.4775	0.3347	0.059*	0.567 (3)
C3CA	0.6975 (2)	0.6728 (2)	0.3129 (2)	0.0286 (7)	0.567 (3)
H3C3	0.7560	0.6695	0.3319	0.034*	0.567 (3)
H3C4	0.6424	0.6543	0.3719	0.034*	0.567 (3)
C4CA	0.6558 (9)	0.7806 (7)	0.2589 (7)	0.0307 (17)	0.567 (3)
H4C4	0.7103	0.8000	0.2009	0.046*	0.567 (3)
H4C5	0.6338	0.8262	0.2995	0.046*	0.567 (3)
H4C6	0.5965	0.7850	0.2415	0.046*	0.567 (3)
C5CA	0.8318 (2)	0.6070 (3)	0.1833 (2)	0.0359 (8)	0.567 (3)
H5C3	0.8891	0.5724	0.2128	0.043*	0.567 (3)
H5C4	0.8303	0.6793	0.1563	0.043*	0.567 (3)
C6CA	0.8528 (10)	0.5612 (7)	0.1033 (5)	0.0468 (19)	0.567 (3)
H6C4	0.8599	0.4885	0.1289	0.070*	0.567 (3)
H6C5	0.9171	0.5720	0.0539	0.070*	0.567 (3)
H6C6	0.7951	0.5935	0.0755	0.070*	0.567 (3)
N1D	0.7016 (5)	0.3706 (6)	1.0068 (3)	0.0238 (9)	0.497 (3)
C1D	0.6422 (3)	0.4484 (3)	0.9448 (3)	0.0369 (9)	0.497 (3)
H1D1	0.6234	0.5137	0.9597	0.044*	0.497 (3)
H1D2	0.5770	0.4322	0.9592	0.044*	0.497 (3)
C2D	0.6979 (5)	0.4605 (4)	0.8397 (4)	0.0554 (14)	0.497 (3)
H2D1	0.7587	0.4838	0.8232	0.083*	0.497 (3)
H2D2	0.6510	0.5101	0.8029	0.083*	0.497 (3)
H2D3	0.7203	0.3953	0.8248	0.083*	0.497 (3)
C3D	0.6371 (4)	0.3527 (3)	1.1070 (3)	0.0395 (10)	0.497 (3)
H3D1	0.6761	0.2907	1.1443	0.047*	0.497 (3)
H3D2	0.5743	0.3391	1.1102	0.047*	0.497 (3)
C4D	0.6026 (4)	0.4366 (3)	1.1557 (3)	0.0509 (12)	0.497 (3)
H4D1	0.6630	0.4437	1.1628	0.076*	0.497 (3)
H4D2	0.5532	0.4200	1.2188	0.076*	0.497 (3)
H4D3	0.5692	0.5004	1.1169	0.076*	0.497 (3)
C5D	0.7959 (5)	0.3981 (8)	0.9893 (7)	0.056 (4)	0.497 (3)
H5D1	0.8162	0.3669	1.0490	0.067*	0.497 (3)
H5D2	0.7756	0.4722	0.9797	0.067*	0.497 (3)
C6D	0.8967 (2)	0.3713 (3)	0.9051 (3)	0.0386 (9)	0.497 (3)
H6D1	0.9136	0.3001	0.9067	0.058*	0.497 (3)
H6D2	0.9546	0.3831	0.9123	0.058*	0.497 (3)
H6D3	0.8845	0.4141	0.8440	0.058*	0.497 (3)
N1DA	0.7094 (5)	0.3795 (6)	0.9764 (3)	0.0238 (9)	0.503 (3)
C1DA	0.6889 (3)	0.4252 (3)	0.8834 (3)	0.0280 (8)	0.503 (3)
H1D3	0.6261	0.4101	0.8888	0.034*	0.503 (3)
H1D4	0.7475	0.3912	0.8365	0.034*	0.503 (3)
C2DA	0.6733 (3)	0.5392 (2)	0.8428 (3)	0.0366 (9)	0.503 (3)
H2D4	0.6176	0.5744	0.8892	0.055*	0.503 (3)
H2D5	0.6548	0.5611	0.7835	0.055*	0.503 (3)
H2D6	0.7377	0.5551	0.8296	0.055*	0.503 (3)
C3DA	0.6148 (3)	0.4082 (3)	1.0529 (3)	0.0336 (8)	0.503 (3)
H3D3	0.5569	0.3955	1.0455	0.040*	0.503 (3)
H3D4	0.5973	0.4817	1.0456	0.040*	0.503 (3)
C4DA	0.6240 (4)	0.3518 (4)	1.1529 (3)	0.0454 (11)	0.503 (3)
H4D4	0.6519	0.2791	1.1577	0.068*	0.503 (3)
H4D5	0.5554	0.3651	1.1996	0.068*	0.503 (3)
H4D6	0.6707	0.3748	1.1658	0.068*	0.503 (3)
C5DA	0.7983 (5)	0.3993 (5)	0.9831 (4)	0.025 (2)	0.503 (3)
H5D3	0.7715	0.4619	1.0048	0.030*	0.503 (3)
H5D4	0.8463	0.4135	0.9179	0.030*	0.503 (3)
C6DA	0.8645 (4)	0.3148 (4)	1.0504 (5)	0.085 (2)	0.503 (3)
H6D4	0.8256	0.3145	1.1172	0.127*	0.503 (3)
H6D5	0.9303	0.3293	1.0345	0.127*	0.503 (3)
H6D6	0.8784	0.2485	1.0408	0.127*	0.503 (3)

### Atomic displacement parameters (Å^2^)

**Table:**

	*U*^11^	*U*^22^	*U*^33^	*U*^12^	*U*^13^	*U*^23^
Cl1	0.02612 (17)	0.0363 (2)	0.01956 (17)	−0.00544 (15)	−0.00797 (13)	−0.00754 (14)
Cl2	0.0435 (2)	0.02378 (18)	0.02120 (17)	−0.00960 (16)	−0.01303 (15)	−0.00132 (14)
O1	0.0167 (4)	0.0196 (5)	0.0260 (5)	−0.0077 (4)	−0.0024 (4)	−0.0100 (4)
C1	0.0167 (6)	0.0149 (6)	0.0184 (6)	−0.0050 (5)	−0.0030 (5)	−0.0072 (5)
C2	0.0169 (6)	0.0189 (7)	0.0210 (7)	−0.0038 (5)	−0.0049 (5)	−0.0077 (5)
C3	0.0224 (6)	0.0214 (7)	0.0175 (6)	−0.0059 (5)	−0.0064 (5)	−0.0061 (5)
C4	0.0188 (6)	0.0226 (7)	0.0197 (7)	−0.0045 (5)	−0.0018 (5)	−0.0098 (5)
C5	0.0164 (6)	0.0193 (7)	0.0231 (7)	−0.0027 (5)	−0.0042 (5)	−0.0088 (5)
C6	0.0179 (6)	0.0138 (6)	0.0209 (6)	−0.0041 (5)	−0.0058 (5)	−0.0061 (5)
C7	0.0192 (6)	0.0141 (6)	0.0215 (7)	−0.0053 (5)	−0.0060 (5)	−0.0056 (5)
C8	0.0214 (6)	0.0199 (7)	0.0259 (7)	−0.0036 (5)	−0.0085 (5)	−0.0061 (6)
C9	0.0285 (7)	0.0195 (7)	0.0281 (8)	−0.0039 (6)	−0.0144 (6)	−0.0036 (6)
C10	0.0331 (7)	0.0169 (7)	0.0187 (7)	−0.0096 (6)	−0.0100 (6)	−0.0017 (5)
C11	0.0239 (6)	0.0164 (6)	0.0195 (6)	−0.0085 (5)	−0.0045 (5)	−0.0047 (5)
C12	0.0201 (6)	0.0131 (6)	0.0209 (6)	−0.0064 (5)	−0.0064 (5)	−0.0048 (5)
C13	0.0160 (6)	0.0164 (6)	0.0175 (6)	−0.0058 (5)	−0.0034 (5)	−0.0057 (5)
C14	0.0200 (6)	0.0165 (6)	0.0152 (6)	−0.0047 (5)	−0.0028 (5)	−0.0047 (5)
C15	0.0210 (6)	0.0214 (7)	0.0208 (7)	−0.0051 (5)	−0.0039 (5)	−0.0056 (5)
C16	0.0228 (7)	0.0272 (8)	0.0287 (8)	−0.0016 (6)	−0.0022 (6)	−0.0102 (6)
C17	0.0333 (8)	0.0233 (8)	0.0383 (9)	0.0001 (6)	−0.0033 (7)	−0.0168 (7)
C18	0.0343 (8)	0.0215 (8)	0.0348 (9)	−0.0079 (6)	−0.0062 (7)	−0.0131 (6)
C19	0.0239 (7)	0.0176 (7)	0.0207 (7)	−0.0060 (5)	−0.0043 (5)	−0.0061 (5)
C20	0.0251 (7)	0.0140 (6)	0.0236 (7)	−0.0086 (5)	−0.0049 (5)	−0.0064 (5)
C21	0.0290 (7)	0.0180 (7)	0.0205 (7)	−0.0113 (6)	−0.0078 (5)	−0.0036 (5)
C22	0.0279 (7)	0.0198 (7)	0.0191 (7)	−0.0127 (6)	−0.0020 (5)	−0.0059 (5)
Cl1A	0.03125 (19)	0.0573 (3)	0.01955 (18)	−0.02149 (19)	−0.00780 (14)	−0.00025 (17)
Cl2A	0.0673 (3)	0.0322 (2)	0.0268 (2)	0.0014 (2)	−0.0264 (2)	−0.01023 (17)
O1A	0.0177 (4)	0.0138 (5)	0.0242 (5)	−0.0014 (4)	−0.0060 (4)	−0.0075 (4)
C1A	0.0158 (6)	0.0128 (6)	0.0202 (6)	−0.0029 (5)	−0.0052 (5)	−0.0055 (5)
C2A	0.0175 (6)	0.0183 (6)	0.0211 (7)	−0.0053 (5)	−0.0067 (5)	−0.0048 (5)
C3A	0.0238 (7)	0.0246 (7)	0.0182 (7)	−0.0100 (6)	−0.0067 (5)	−0.0026 (5)
C4A	0.0197 (6)	0.0255 (7)	0.0236 (7)	−0.0098 (6)	−0.0026 (5)	−0.0042 (6)
C5A	0.0167 (6)	0.0193 (7)	0.0291 (7)	−0.0060 (5)	−0.0076 (5)	−0.0055 (6)
C6A	0.0189 (6)	0.0130 (6)	0.0244 (7)	−0.0033 (5)	−0.0091 (5)	−0.0061 (5)
C7A	0.0224 (6)	0.0124 (6)	0.0262 (7)	−0.0022 (5)	−0.0123 (5)	−0.0058 (5)
C8A	0.0278 (7)	0.0204 (7)	0.0374 (9)	−0.0027 (6)	−0.0198 (6)	−0.0073 (6)
C9A	0.0416 (9)	0.0212 (7)	0.0394 (9)	0.0005 (6)	−0.0295 (8)	−0.0088 (7)
C10A	0.0446 (9)	0.0183 (7)	0.0250 (7)	0.0008 (6)	−0.0199 (7)	−0.0079 (6)
C11A	0.0312 (7)	0.0175 (7)	0.0231 (7)	0.0004 (6)	−0.0118 (6)	−0.0086 (6)
C12A	0.0232 (6)	0.0125 (6)	0.0221 (7)	−0.0007 (5)	−0.0106 (5)	−0.0073 (5)
C13A	0.0164 (6)	0.0142 (6)	0.0176 (6)	−0.0014 (5)	−0.0056 (5)	−0.0058 (5)
C14A	0.0180 (6)	0.0169 (6)	0.0138 (6)	−0.0047 (5)	−0.0038 (5)	−0.0040 (5)
C15A	0.0189 (6)	0.0185 (7)	0.0206 (7)	−0.0027 (5)	−0.0053 (5)	−0.0064 (5)
C16A	0.0176 (6)	0.0275 (8)	0.0260 (7)	−0.0066 (6)	−0.0041 (5)	−0.0064 (6)
C17A	0.0237 (7)	0.0242 (8)	0.0319 (8)	−0.0122 (6)	−0.0046 (6)	−0.0044 (6)
C18A	0.0238 (7)	0.0172 (7)	0.0281 (7)	−0.0067 (5)	−0.0059 (6)	−0.0041 (6)
C19A	0.0184 (6)	0.0175 (6)	0.0159 (6)	−0.0044 (5)	−0.0043 (5)	−0.0046 (5)
C20A	0.0180 (6)	0.0119 (6)	0.0201 (6)	−0.0032 (5)	−0.0047 (5)	−0.0061 (5)
C21A	0.0219 (6)	0.0147 (6)	0.0185 (6)	−0.0031 (5)	−0.0074 (5)	−0.0042 (5)
C22A	0.0214 (6)	0.0162 (6)	0.0161 (6)	−0.0045 (5)	−0.0033 (5)	−0.0049 (5)
N1B	0.0200 (5)	0.0185 (6)	0.0241 (6)	−0.0034 (4)	−0.0066 (5)	−0.0084 (5)
C1B	0.0227 (7)	0.0299 (8)	0.0300 (8)	−0.0084 (6)	−0.0037 (6)	−0.0122 (6)
C2B	0.0356 (8)	0.0369 (9)	0.0271 (8)	−0.0120 (7)	−0.0035 (7)	−0.0101 (7)
C3B	0.0253 (7)	0.0212 (7)	0.0257 (7)	−0.0033 (6)	−0.0096 (6)	−0.0079 (6)
C4B	0.0301 (8)	0.0315 (8)	0.0323 (8)	−0.0045 (6)	−0.0114 (6)	−0.0161 (7)
C5B	0.0242 (7)	0.0189 (7)	0.0280 (7)	−0.0022 (5)	−0.0090 (6)	−0.0078 (6)
C6B	0.0223 (7)	0.0279 (8)	0.0356 (9)	−0.0011 (6)	−0.0078 (6)	−0.0110 (7)
N1C	0.0219 (12)	0.0267 (8)	0.0237 (19)	−0.0050 (8)	−0.0074 (13)	−0.0075 (9)
C1C	0.035 (2)	0.038 (3)	0.026 (2)	−0.009 (2)	−0.0104 (18)	−0.0003 (19)
C2C	0.034 (4)	0.055 (5)	0.038 (3)	−0.019 (3)	−0.008 (3)	0.005 (3)
C3C	0.0278 (17)	0.031 (2)	0.032 (2)	−0.0087 (15)	−0.0125 (15)	−0.0090 (15)
C4C	0.032 (3)	0.035 (5)	0.036 (4)	−0.007 (3)	−0.014 (3)	−0.015 (4)
C5C	0.0206 (15)	0.029 (2)	0.0225 (19)	−0.0028 (13)	−0.0084 (13)	−0.0057 (14)
C6C	0.034 (3)	0.035 (3)	0.023 (4)	−0.004 (3)	−0.008 (3)	−0.007 (3)
N1CA	0.0219 (12)	0.0267 (8)	0.0237 (19)	−0.0050 (8)	−0.0074 (13)	−0.0075 (9)
C1CA	0.0298 (17)	0.0235 (18)	0.046 (2)	−0.0022 (13)	−0.0211 (15)	−0.0059 (14)
C2CA	0.041 (3)	0.034 (2)	0.041 (3)	−0.017 (2)	−0.011 (3)	−0.0003 (17)
C3CA	0.0290 (13)	0.0302 (16)	0.0298 (15)	−0.0045 (11)	−0.0131 (11)	−0.0103 (12)
C4CA	0.029 (2)	0.025 (3)	0.039 (4)	−0.0059 (17)	−0.015 (2)	−0.005 (2)
C5CA	0.0266 (14)	0.046 (2)	0.0378 (17)	−0.0115 (13)	−0.0037 (12)	−0.0185 (14)
C6CA	0.039 (3)	0.060 (5)	0.041 (5)	−0.013 (3)	−0.005 (4)	−0.021 (4)
N1D	0.0213 (10)	0.0215 (14)	0.030 (3)	−0.0069 (8)	−0.0059 (19)	−0.009 (2)
C1D	0.0354 (18)	0.0319 (19)	0.041 (2)	0.0001 (15)	−0.0150 (16)	−0.0126 (16)
C2D	0.083 (4)	0.046 (3)	0.040 (3)	−0.020 (3)	−0.021 (3)	−0.008 (2)
C3D	0.061 (3)	0.036 (2)	0.029 (2)	−0.028 (2)	−0.001 (2)	−0.014 (2)
C4D	0.075 (3)	0.048 (3)	0.038 (2)	−0.033 (2)	0.001 (2)	−0.0221 (18)
C5D	0.018 (4)	0.058 (6)	0.112 (7)	−0.004 (4)	−0.012 (4)	−0.060 (5)
C6D	0.0235 (15)	0.0343 (19)	0.060 (2)	−0.0085 (14)	−0.0069 (15)	−0.0190 (17)
N1DA	0.0213 (10)	0.0215 (14)	0.030 (3)	−0.0069 (8)	−0.0059 (19)	−0.009 (2)
C1DA	0.0350 (18)	0.0208 (18)	0.033 (2)	−0.0121 (14)	−0.0138 (18)	−0.0029 (16)
C2DA	0.049 (2)	0.0192 (17)	0.048 (2)	−0.0123 (14)	−0.0269 (17)	0.0003 (14)
C3DA	0.0309 (16)	0.0271 (18)	0.039 (2)	−0.0080 (14)	−0.0002 (14)	−0.0149 (17)
C4DA	0.061 (3)	0.045 (3)	0.028 (2)	−0.024 (2)	0.004 (2)	−0.016 (2)
C5DA	0.034 (5)	0.019 (3)	0.026 (2)	−0.013 (3)	−0.012 (2)	0.000 (2)
C6DA	0.072 (3)	0.054 (3)	0.165 (7)	0.014 (3)	−0.083 (4)	−0.047 (4)

### Geometric parameters (Å, º)

**Table:**

Cl1—C3	1.7427 (14)	C3B—H3B1	0.9900
Cl2—C10	1.7502 (15)	C3B—H3B2	0.9900
O1—C13	1.4229 (15)	C4B—H4B1	0.9800
O1—H1	0.8400	C4B—H4B2	0.9800
C1—C2	1.3804 (19)	C4B—H4B3	0.9800
C1—C6	1.4009 (18)	C5B—C6B	1.516 (2)
C1—C13	1.5282 (18)	C5B—H5B1	0.9900
C2—C3	1.3951 (19)	C5B—H5B2	0.9900
C2—H2	0.9500	C6B—H6B1	0.9800
C3—C4	1.3945 (19)	C6B—H6B2	0.9800
C4—C5	1.391 (2)	C6B—H6B3	0.9800
C4—H4	0.9500	N1C—C5C	1.466 (8)
C5—C6	1.3893 (18)	N1C—C1C	1.471 (8)
C5—H5	0.9500	N1C—C3C	1.473 (8)
C6—C7	1.4709 (19)	C1C—C2C	1.515 (8)
C7—C8	1.3906 (19)	C1C—H1C1	0.9900
C7—C12	1.4017 (18)	C1C—H1C2	0.9900
C8—C9	1.394 (2)	C2C—H2C1	0.9800
C8—H8	0.9500	C2C—H2C2	0.9800
C9—C10	1.387 (2)	C2C—H2C3	0.9800
C9—H9	0.9500	C3C—C4C	1.516 (9)
C10—C11	1.392 (2)	C3C—H3C1	0.9900
C11—C12	1.3782 (19)	C3C—H3C2	0.9900
C11—H11	0.9500	C4C—H4C1	0.9800
C12—C13	1.5296 (18)	C4C—H4C2	0.9800
C13—C14	1.5369 (19)	C4C—H4C3	0.9800
C14—C15	1.3945 (19)	C5C—C6C	1.521 (7)
C14—C19	1.4109 (19)	C5C—H5C1	0.9900
C15—C16	1.388 (2)	C5C—H5C2	0.9900
C15—H15	0.9500	C6C—H6C1	0.9800
C16—C17	1.380 (2)	C6C—H6C2	0.9800
C16—H16	0.9500	C6C—H6C3	0.9800
C17—C18	1.384 (2)	N1CA—C1CA	1.467 (6)
C17—H17	0.9500	N1CA—C5CA	1.470 (7)
C18—C19	1.398 (2)	N1CA—C3CA	1.470 (6)
C18—H18	0.9500	C1CA—C2CA	1.516 (5)
C19—C20	1.4935 (19)	C1CA—H1C3	0.9900
C20—C21	1.396 (2)	C1CA—H1C4	0.9900
C20—C22^i^	1.398 (2)	C2CA—H2C4	0.9800
C21—C22	1.387 (2)	C2CA—H2C5	0.9800
C21—H21	0.9500	C2CA—H2C6	0.9800
C22—C20^i^	1.398 (2)	C3CA—C4CA	1.519 (7)
C22—H22	0.9500	C3CA—H3C3	0.9900
Cl1A—C3A	1.7383 (15)	C3CA—H3C4	0.9900
Cl2A—C10A	1.7408 (16)	C4CA—H4C4	0.9800
O1A—C13A	1.4243 (15)	C4CA—H4C5	0.9800
O1A—H1A	0.8400	C4CA—H4C6	0.9800
C1A—C2A	1.3777 (19)	C5CA—C6CA	1.522 (7)
C1A—C6A	1.4022 (17)	C5CA—H5C3	0.9900
C1A—C13A	1.5252 (18)	C5CA—H5C4	0.9900
C2A—C3A	1.3905 (19)	C6CA—H6C4	0.9800
C2A—H2A	0.9500	C6CA—H6C5	0.9800
C3A—C4A	1.395 (2)	C6CA—H6C6	0.9800
C4A—C5A	1.391 (2)	N1D—C1D	1.452 (6)
C4A—H4A	0.9500	N1D—C3D	1.472 (6)
C5A—C6A	1.3877 (19)	N1D—C5D	1.478 (7)
C5A—H5A	0.9500	C1D—C2D	1.508 (5)
C6A—C7A	1.4693 (19)	C1D—H1D1	0.9900
C7A—C8A	1.3937 (19)	C1D—H1D2	0.9900
C7A—C12A	1.3983 (19)	C2D—H2D1	0.9800
C8A—C9A	1.390 (2)	C2D—H2D2	0.9800
C8A—H8A	0.9500	C2D—H2D3	0.9800
C9A—C10A	1.382 (2)	C3D—C4D	1.520 (5)
C9A—H9A	0.9500	C3D—H3D1	0.9900
C10A—C11A	1.395 (2)	C3D—H3D2	0.9900
C11A—C12A	1.378 (2)	C4D—H4D1	0.9800
C11A—H11A	0.9500	C4D—H4D2	0.9800
C12A—C13A	1.5311 (18)	C4D—H4D3	0.9800
C13A—C14A	1.5308 (18)	C5D—C6D	1.579 (7)
C14A—C15A	1.3978 (18)	C5D—H5D1	0.9900
C14A—C19A	1.4100 (19)	C5D—H5D2	0.9900
C15A—C16A	1.389 (2)	C6D—H6D1	0.9800
C15A—H15A	0.9500	C6D—H6D2	0.9800
C16A—C17A	1.380 (2)	C6D—H6D3	0.9800
C16A—H16A	0.9500	N1DA—C1DA	1.469 (6)
C17A—C18A	1.391 (2)	N1DA—C3DA	1.471 (6)
C17A—H17A	0.9500	N1DA—C5DA	1.484 (7)
C18A—C19A	1.3964 (19)	C1DA—C2DA	1.529 (4)
C18A—H18A	0.9500	C1DA—H1D3	0.9900
C19A—C20A	1.4979 (18)	C1DA—H1D4	0.9900
C20A—C22A^ii^	1.3956 (19)	C2DA—H2D4	0.9800
C20A—C21A	1.3966 (19)	C2DA—H2D5	0.9800
C21A—C22A	1.3891 (19)	C2DA—H2D6	0.9800
C21A—H21A	0.9500	C3DA—C4DA	1.521 (5)
C22A—C20A^ii^	1.3956 (19)	C3DA—H3D3	0.9900
C22A—H22A	0.9500	C3DA—H3D4	0.9900
N1B—C1B	1.4714 (18)	C4DA—H4D4	0.9800
N1B—C5B	1.4754 (18)	C4DA—H4D5	0.9800
N1B—C3B	1.4809 (19)	C4DA—H4D6	0.9800
C1B—C2B	1.519 (2)	C5DA—C6DA	1.589 (7)
C1B—H1B1	0.9900	C5DA—H5D3	0.9900
C1B—H1B2	0.9900	C5DA—H5D4	0.9900
C2B—H2B1	0.9800	C6DA—H6D4	0.9800
C2B—H2B2	0.9800	C6DA—H6D5	0.9800
C2B—H2B3	0.9800	C6DA—H6D6	0.9800
C3B—C4B	1.527 (2)		
			
C13—O1—H1	109.5	N1B—C5B—H5B1	108.9
C2—C1—C6	121.32 (12)	C6B—C5B—H5B1	108.9
C2—C1—C13	127.78 (12)	N1B—C5B—H5B2	108.9
C6—C1—C13	110.87 (11)	C6B—C5B—H5B2	108.9
C1—C2—C3	117.59 (12)	H5B1—C5B—H5B2	107.7
C1—C2—H2	121.2	C5B—C6B—H6B1	109.5
C3—C2—H2	121.2	C5B—C6B—H6B2	109.5
C4—C3—C2	122.09 (13)	H6B1—C6B—H6B2	109.5
C4—C3—Cl1	119.22 (11)	C5B—C6B—H6B3	109.5
C2—C3—Cl1	118.69 (11)	H6B1—C6B—H6B3	109.5
C5—C4—C3	119.43 (12)	H6B2—C6B—H6B3	109.5
C5—C4—H4	120.3	C5C—N1C—C1C	109.4 (7)
C3—C4—H4	120.3	C5C—N1C—C3C	110.0 (7)
C6—C5—C4	119.23 (12)	C1C—N1C—C3C	110.4 (7)
C6—C5—H5	120.4	N1C—C1C—C2C	113.1 (7)
C4—C5—H5	120.4	N1C—C1C—H1C1	109.0
C5—C6—C1	120.30 (13)	C2C—C1C—H1C1	109.0
C5—C6—C7	130.96 (12)	N1C—C1C—H1C2	109.0
C1—C6—C7	108.71 (11)	C2C—C1C—H1C2	109.0
C8—C7—C12	120.11 (13)	H1C1—C1C—H1C2	107.8
C8—C7—C6	131.60 (13)	C1C—C2C—H2C1	109.5
C12—C7—C6	108.27 (12)	C1C—C2C—H2C2	109.5
C7—C8—C9	119.29 (13)	H2C1—C2C—H2C2	109.5
C7—C8—H8	120.4	C1C—C2C—H2C3	109.5
C9—C8—H8	120.4	H2C1—C2C—H2C3	109.5
C10—C9—C8	119.12 (13)	H2C2—C2C—H2C3	109.5
C10—C9—H9	120.4	N1C—C3C—C4C	113.3 (8)
C8—C9—H9	120.4	N1C—C3C—H3C1	108.9
C9—C10—C11	122.68 (13)	C4C—C3C—H3C1	108.9
C9—C10—Cl2	118.86 (11)	N1C—C3C—H3C2	108.9
C11—C10—Cl2	118.44 (11)	C4C—C3C—H3C2	108.9
C12—C11—C10	117.41 (13)	H3C1—C3C—H3C2	107.7
C12—C11—H11	121.3	C3C—C4C—H4C1	109.5
C10—C11—H11	121.3	C3C—C4C—H4C2	109.5
C11—C12—C7	121.39 (13)	H4C1—C4C—H4C2	109.5
C11—C12—C13	127.54 (12)	C3C—C4C—H4C3	109.5
C7—C12—C13	111.07 (12)	H4C1—C4C—H4C3	109.5
O1—C13—C1	111.11 (10)	H4C2—C4C—H4C3	109.5
O1—C13—C12	111.35 (11)	N1C—C5C—C6C	112.6 (7)
C1—C13—C12	101.02 (10)	N1C—C5C—H5C1	109.1
O1—C13—C14	107.02 (10)	C6C—C5C—H5C1	109.1
C1—C13—C14	114.40 (11)	N1C—C5C—H5C2	109.1
C12—C13—C14	111.99 (11)	C6C—C5C—H5C2	109.1
C15—C14—C19	118.90 (13)	H5C1—C5C—H5C2	107.8
C15—C14—C13	118.26 (12)	C5C—C6C—H6C1	109.5
C19—C14—C13	122.80 (12)	C5C—C6C—H6C2	109.5
C16—C15—C14	121.50 (14)	H6C1—C6C—H6C2	109.5
C16—C15—H15	119.2	C5C—C6C—H6C3	109.5
C14—C15—H15	119.2	H6C1—C6C—H6C3	109.5
C17—C16—C15	119.80 (14)	H6C2—C6C—H6C3	109.5
C17—C16—H16	120.1	C1CA—N1CA—C5CA	110.5 (6)
C15—C16—H16	120.1	C1CA—N1CA—C3CA	110.3 (5)
C16—C17—C18	119.45 (15)	C5CA—N1CA—C3CA	110.3 (5)
C16—C17—H17	120.3	N1CA—C1CA—C2CA	113.7 (5)
C18—C17—H17	120.3	N1CA—C1CA—H1C3	108.8
C17—C18—C19	121.92 (15)	C2CA—C1CA—H1C3	108.8
C17—C18—H18	119.0	N1CA—C1CA—H1C4	108.8
C19—C18—H18	119.0	C2CA—C1CA—H1C4	108.8
C18—C19—C14	118.42 (13)	H1C3—C1CA—H1C4	107.7
C18—C19—C20	117.00 (13)	C1CA—C2CA—H2C4	109.5
C14—C19—C20	124.57 (12)	C1CA—C2CA—H2C5	109.5
C21—C20—C22^i^	118.20 (13)	H2C4—C2CA—H2C5	109.5
C21—C20—C19	121.51 (13)	C1CA—C2CA—H2C6	109.5
C22^i^—C20—C19	120.16 (13)	H2C4—C2CA—H2C6	109.5
C22—C21—C20	120.84 (13)	H2C5—C2CA—H2C6	109.5
C22—C21—H21	119.6	N1CA—C3CA—C4CA	113.5 (6)
C20—C21—H21	119.6	N1CA—C3CA—H3C3	108.9
C21—C22—C20^i^	120.95 (13)	C4CA—C3CA—H3C3	108.9
C21—C22—H22	119.5	N1CA—C3CA—H3C4	108.9
C20^i^—C22—H22	119.5	C4CA—C3CA—H3C4	108.9
C13A—O1A—H1A	109.5	H3C3—C3CA—H3C4	107.7
C2A—C1A—C6A	121.37 (12)	C3CA—C4CA—H4C4	109.5
C2A—C1A—C13A	127.42 (11)	C3CA—C4CA—H4C5	109.5
C6A—C1A—C13A	111.20 (12)	H4C4—C4CA—H4C5	109.5
C1A—C2A—C3A	117.54 (12)	C3CA—C4CA—H4C6	109.5
C1A—C2A—H2A	121.2	H4C4—C4CA—H4C6	109.5
C3A—C2A—H2A	121.2	H4C5—C4CA—H4C6	109.5
C2A—C3A—C4A	122.28 (13)	N1CA—C5CA—C6CA	112.3 (6)
C2A—C3A—Cl1A	118.45 (11)	N1CA—C5CA—H5C3	109.1
C4A—C3A—Cl1A	119.26 (11)	C6CA—C5CA—H5C3	109.1
C5A—C4A—C3A	119.30 (13)	N1CA—C5CA—H5C4	109.1
C5A—C4A—H4A	120.4	C6CA—C5CA—H5C4	109.1
C3A—C4A—H4A	120.4	H5C3—C5CA—H5C4	107.9
C6A—C5A—C4A	119.19 (12)	C5CA—C6CA—H6C4	109.5
C6A—C5A—H5A	120.4	C5CA—C6CA—H6C5	109.5
C4A—C5A—H5A	120.4	H6C4—C6CA—H6C5	109.5
C5A—C6A—C1A	120.27 (13)	C5CA—C6CA—H6C6	109.5
C5A—C6A—C7A	131.30 (12)	H6C4—C6CA—H6C6	109.5
C1A—C6A—C7A	108.37 (12)	H6C5—C6CA—H6C6	109.5
C8A—C7A—C12A	120.06 (14)	C1D—N1D—C3D	111.1 (5)
C8A—C7A—C6A	131.51 (14)	C1D—N1D—C5D	108.6 (6)
C12A—C7A—C6A	108.43 (11)	C3D—N1D—C5D	114.6 (5)
C9A—C8A—C7A	118.70 (15)	N1D—C1D—C2D	114.4 (4)
C9A—C8A—H8A	120.7	N1D—C1D—H1D1	108.7
C7A—C8A—H8A	120.7	C2D—C1D—H1D1	108.7
C10A—C9A—C8A	120.05 (14)	N1D—C1D—H1D2	108.7
C10A—C9A—H9A	120.0	C2D—C1D—H1D2	108.7
C8A—C9A—H9A	120.0	H1D1—C1D—H1D2	107.6
C9A—C10A—C11A	122.21 (15)	C1D—C2D—H2D1	109.5
C9A—C10A—Cl2A	118.90 (12)	C1D—C2D—H2D2	109.5
C11A—C10A—Cl2A	118.87 (13)	H2D1—C2D—H2D2	109.5
C12A—C11A—C10A	117.19 (15)	C1D—C2D—H2D3	109.5
C12A—C11A—H11A	121.4	H2D1—C2D—H2D3	109.5
C10A—C11A—H11A	121.4	H2D2—C2D—H2D3	109.5
C11A—C12A—C7A	121.76 (13)	N1D—C3D—C4D	117.0 (5)
C11A—C12A—C13A	127.11 (13)	N1D—C3D—H3D1	108.1
C7A—C12A—C13A	111.13 (12)	C4D—C3D—H3D1	108.1
O1A—C13A—C1A	111.32 (10)	N1D—C3D—H3D2	108.1
O1A—C13A—C14A	107.22 (10)	C4D—C3D—H3D2	108.1
C1A—C13A—C14A	114.25 (11)	H3D1—C3D—H3D2	107.3
O1A—C13A—C12A	110.73 (10)	C3D—C4D—H4D1	109.5
C1A—C13A—C12A	100.74 (10)	C3D—C4D—H4D2	109.5
C14A—C13A—C12A	112.58 (11)	H4D1—C4D—H4D2	109.5
C15A—C14A—C19A	118.83 (12)	C3D—C4D—H4D3	109.5
C15A—C14A—C13A	118.53 (12)	H4D1—C4D—H4D3	109.5
C19A—C14A—C13A	122.59 (11)	H4D2—C4D—H4D3	109.5
C16A—C15A—C14A	121.32 (13)	N1D—C5D—C6D	121.3 (7)
C16A—C15A—H15A	119.3	N1D—C5D—H5D1	107.0
C14A—C15A—H15A	119.3	C6D—C5D—H5D1	107.0
C17A—C16A—C15A	119.96 (13)	N1D—C5D—H5D2	107.0
C17A—C16A—H16A	120.0	C6D—C5D—H5D2	107.0
C15A—C16A—H16A	120.0	H5D1—C5D—H5D2	106.7
C16A—C17A—C18A	119.45 (13)	C5D—C6D—H6D1	109.5
C16A—C17A—H17A	120.3	C5D—C6D—H6D2	109.5
C18A—C17A—H17A	120.3	H6D1—C6D—H6D2	109.5
C17A—C18A—C19A	121.57 (14)	C5D—C6D—H6D3	109.5
C17A—C18A—H18A	119.2	H6D1—C6D—H6D3	109.5
C19A—C18A—H18A	119.2	H6D2—C6D—H6D3	109.5
C18A—C19A—C14A	118.79 (12)	C1DA—N1DA—C3DA	110.3 (5)
C18A—C19A—C20A	116.71 (12)	C1DA—N1DA—C5DA	115.5 (5)
C14A—C19A—C20A	124.50 (12)	C3DA—N1DA—C5DA	110.9 (5)
C22A^ii^—C20A—C21A	118.27 (12)	N1DA—C1DA—C2DA	117.3 (4)
C22A^ii^—C20A—C19A	120.83 (12)	N1DA—C1DA—H1D3	108.0
C21A—C20A—C19A	120.78 (12)	C2DA—C1DA—H1D3	108.0
C22A—C21A—C20A	120.81 (13)	N1DA—C1DA—H1D4	108.0
C22A—C21A—H21A	119.6	C2DA—C1DA—H1D4	108.0
C20A—C21A—H21A	119.6	H1D3—C1DA—H1D4	107.2
C21A—C22A—C20A^ii^	120.92 (12)	C1DA—C2DA—H2D4	109.5
C21A—C22A—H22A	119.5	C1DA—C2DA—H2D5	109.5
C20A^ii^—C22A—H22A	119.5	H2D4—C2DA—H2D5	109.5
C1B—N1B—C5B	111.33 (12)	C1DA—C2DA—H2D6	109.5
C1B—N1B—C3B	110.96 (11)	H2D4—C2DA—H2D6	109.5
C5B—N1B—C3B	113.05 (11)	H2D5—C2DA—H2D6	109.5
N1B—C1B—C2B	113.88 (13)	N1DA—C3DA—C4DA	113.9 (4)
N1B—C1B—H1B1	108.8	N1DA—C3DA—H3D3	108.8
C2B—C1B—H1B1	108.8	C4DA—C3DA—H3D3	108.8
N1B—C1B—H1B2	108.8	N1DA—C3DA—H3D4	108.8
C2B—C1B—H1B2	108.8	C4DA—C3DA—H3D4	108.8
H1B1—C1B—H1B2	107.7	H3D3—C3DA—H3D4	107.7
C1B—C2B—H2B1	109.5	C3DA—C4DA—H4D4	109.5
C1B—C2B—H2B2	109.5	C3DA—C4DA—H4D5	109.5
H2B1—C2B—H2B2	109.5	H4D4—C4DA—H4D5	109.5
C1B—C2B—H2B3	109.5	C3DA—C4DA—H4D6	109.5
H2B1—C2B—H2B3	109.5	H4D4—C4DA—H4D6	109.5
H2B2—C2B—H2B3	109.5	H4D5—C4DA—H4D6	109.5
N1B—C3B—C4B	116.88 (13)	N1DA—C5DA—C6DA	118.7 (5)
N1B—C3B—H3B1	108.1	N1DA—C5DA—H5D3	107.6
C4B—C3B—H3B1	108.1	C6DA—C5DA—H5D3	107.6
N1B—C3B—H3B2	108.1	N1DA—C5DA—H5D4	107.6
C4B—C3B—H3B2	108.1	C6DA—C5DA—H5D4	107.6
H3B1—C3B—H3B2	107.3	H5D3—C5DA—H5D4	107.1
C3B—C4B—H4B1	109.5	C5DA—C6DA—H6D4	109.5
C3B—C4B—H4B2	109.5	C5DA—C6DA—H6D5	109.5
H4B1—C4B—H4B2	109.5	H6D4—C6DA—H6D5	109.5
C3B—C4B—H4B3	109.5	C5DA—C6DA—H6D6	109.5
H4B1—C4B—H4B3	109.5	H6D4—C6DA—H6D6	109.5
H4B2—C4B—H4B3	109.5	H6D5—C6DA—H6D6	109.5
N1B—C5B—C6B	113.51 (12)		
			
C6—C1—C2—C3	0.5 (2)	C5A—C6A—C7A—C12A	−175.28 (14)
C13—C1—C2—C3	178.35 (13)	C1A—C6A—C7A—C12A	2.01 (15)
C1—C2—C3—C4	1.1 (2)	C12A—C7A—C8A—C9A	0.9 (2)
C1—C2—C3—Cl1	−179.17 (10)	C6A—C7A—C8A—C9A	−178.38 (14)
C2—C3—C4—C5	−1.4 (2)	C7A—C8A—C9A—C10A	0.9 (2)
Cl1—C3—C4—C5	178.80 (11)	C8A—C9A—C10A—C11A	−1.7 (2)
C3—C4—C5—C6	0.2 (2)	C8A—C9A—C10A—Cl2A	176.99 (12)
C4—C5—C6—C1	1.4 (2)	C9A—C10A—C11A—C12A	0.6 (2)
C4—C5—C6—C7	179.40 (13)	Cl2A—C10A—C11A—C12A	−178.09 (11)
C2—C1—C6—C5	−1.8 (2)	C10A—C11A—C12A—C7A	1.3 (2)
C13—C1—C6—C5	−179.92 (12)	C10A—C11A—C12A—C13A	−177.59 (13)
C2—C1—C6—C7	179.81 (12)	C8A—C7A—C12A—C11A	−2.0 (2)
C13—C1—C6—C7	1.66 (15)	C6A—C7A—C12A—C11A	177.39 (12)
C5—C6—C7—C8	0.0 (3)	C8A—C7A—C12A—C13A	177.00 (12)
C1—C6—C7—C8	178.18 (14)	C6A—C7A—C12A—C13A	−3.57 (15)
C5—C6—C7—C12	−178.43 (14)	C2A—C1A—C13A—O1A	−64.61 (17)
C1—C6—C7—C12	−0.24 (15)	C6A—C1A—C13A—O1A	115.17 (12)
C12—C7—C8—C9	0.3 (2)	C2A—C1A—C13A—C14A	57.01 (18)
C6—C7—C8—C9	−177.96 (14)	C6A—C1A—C13A—C14A	−123.21 (12)
C7—C8—C9—C10	−0.3 (2)	C2A—C1A—C13A—C12A	177.95 (13)
C8—C9—C10—C11	−0.3 (2)	C6A—C1A—C13A—C12A	−2.27 (14)
C8—C9—C10—Cl2	177.89 (11)	C11A—C12A—C13A—O1A	64.65 (17)
C9—C10—C11—C12	0.7 (2)	C7A—C12A—C13A—O1A	−114.32 (12)
Cl2—C10—C11—C12	−177.43 (10)	C11A—C12A—C13A—C1A	−177.48 (13)
C10—C11—C12—C7	−0.7 (2)	C7A—C12A—C13A—C1A	3.54 (14)
C10—C11—C12—C13	179.42 (12)	C11A—C12A—C13A—C14A	−55.37 (18)
C8—C7—C12—C11	0.2 (2)	C7A—C12A—C13A—C14A	125.66 (12)
C6—C7—C12—C11	178.82 (12)	O1A—C13A—C14A—C15A	5.45 (16)
C8—C7—C12—C13	−179.90 (12)	C1A—C13A—C14A—C15A	−118.40 (13)
C6—C7—C12—C13	−1.27 (15)	C12A—C13A—C14A—C15A	127.48 (13)
C2—C1—C13—O1	−62.04 (17)	O1A—C13A—C14A—C19A	−171.97 (12)
C6—C1—C13—O1	115.96 (12)	C1A—C13A—C14A—C19A	64.18 (16)
C2—C1—C13—C12	179.74 (13)	C12A—C13A—C14A—C19A	−49.94 (17)
C6—C1—C13—C12	−2.26 (14)	C19A—C14A—C15A—C16A	2.5 (2)
C2—C1—C13—C14	59.26 (18)	C13A—C14A—C15A—C16A	−175.04 (13)
C6—C1—C13—C14	−122.74 (12)	C14A—C15A—C16A—C17A	−0.1 (2)
C11—C12—C13—O1	63.97 (17)	C15A—C16A—C17A—C18A	−1.4 (2)
C7—C12—C13—O1	−115.93 (12)	C16A—C17A—C18A—C19A	0.6 (2)
C11—C12—C13—C1	−177.99 (13)	C17A—C18A—C19A—C14A	1.8 (2)
C7—C12—C13—C1	2.11 (14)	C17A—C18A—C19A—C20A	−177.53 (14)
C11—C12—C13—C14	−55.82 (17)	C15A—C14A—C19A—C18A	−3.2 (2)
C7—C12—C13—C14	124.28 (12)	C13A—C14A—C19A—C18A	174.17 (13)
O1—C13—C14—C15	6.18 (16)	C15A—C14A—C19A—C20A	176.00 (13)
C1—C13—C14—C15	−117.35 (13)	C13A—C14A—C19A—C20A	−6.6 (2)
C12—C13—C14—C15	128.47 (13)	C18A—C19A—C20A—C22A^ii^	−77.30 (17)
O1—C13—C14—C19	−171.45 (12)	C14A—C19A—C20A—C22A^ii^	103.44 (16)
C1—C13—C14—C19	65.02 (17)	C18A—C19A—C20A—C21A	98.52 (16)
C12—C13—C14—C19	−49.16 (17)	C14A—C19A—C20A—C21A	−80.73 (18)
C19—C14—C15—C16	1.4 (2)	C22A^ii^—C20A—C21A—C22A	−0.6 (2)
C13—C14—C15—C16	−176.30 (13)	C19A—C20A—C21A—C22A	−176.54 (13)
C14—C15—C16—C17	−0.6 (2)	C20A—C21A—C22A—C20A^ii^	0.6 (2)
C15—C16—C17—C18	−0.2 (3)	C5B—N1B—C1B—C2B	62.98 (17)
C16—C17—C18—C19	0.1 (3)	C3B—N1B—C1B—C2B	−170.17 (13)
C17—C18—C19—C14	0.8 (2)	C1B—N1B—C3B—C4B	−71.11 (16)
C17—C18—C19—C20	179.49 (15)	C5B—N1B—C3B—C4B	54.78 (17)
C15—C14—C19—C18	−1.5 (2)	C1B—N1B—C5B—C6B	−177.19 (13)
C13—C14—C19—C18	176.09 (13)	C3B—N1B—C5B—C6B	57.12 (16)
C15—C14—C19—C20	179.91 (13)	C5C—N1C—C1C—C2C	−72.5 (10)
C13—C14—C19—C20	−2.5 (2)	C3C—N1C—C1C—C2C	166.3 (7)
C18—C19—C20—C21	102.54 (17)	C5C—N1C—C3C—C4C	174.3 (9)
C14—C19—C20—C21	−78.87 (19)	C1C—N1C—C3C—C4C	−64.8 (11)
C18—C19—C20—C22^i^	−73.40 (18)	C1C—N1C—C5C—C6C	170.4 (8)
C14—C19—C20—C22^i^	105.19 (17)	C3C—N1C—C5C—C6C	−68.2 (11)
C22^i^—C20—C21—C22	−0.8 (2)	C5CA—N1CA—C1CA—C2CA	−161.8 (5)
C19—C20—C21—C22	−176.85 (13)	C3CA—N1CA—C1CA—C2CA	75.9 (8)
C20—C21—C22—C20^i^	0.9 (2)	C1CA—N1CA—C3CA—C4CA	−164.3 (7)
C6A—C1A—C2A—C3A	1.4 (2)	C5CA—N1CA—C3CA—C4CA	73.4 (9)
C13A—C1A—C2A—C3A	−178.87 (13)	C1CA—N1CA—C5CA—C6CA	78.2 (8)
C1A—C2A—C3A—C4A	0.6 (2)	C3CA—N1CA—C5CA—C6CA	−159.6 (6)
C1A—C2A—C3A—Cl1A	−179.27 (11)	C3D—N1D—C1D—C2D	−171.4 (5)
C2A—C3A—C4A—C5A	−1.8 (2)	C5D—N1D—C1D—C2D	61.8 (7)
Cl1A—C3A—C4A—C5A	178.12 (11)	C1D—N1D—C3D—C4D	−69.0 (7)
C3A—C4A—C5A—C6A	0.9 (2)	C5D—N1D—C3D—C4D	54.4 (8)
C4A—C5A—C6A—C1A	1.0 (2)	C1D—N1D—C5D—C6D	−84.2 (9)
C4A—C5A—C6A—C7A	178.05 (14)	C3D—N1D—C5D—C6D	151.0 (7)
C2A—C1A—C6A—C5A	−2.2 (2)	C3DA—N1DA—C1DA—C2DA	72.0 (6)
C13A—C1A—C6A—C5A	177.97 (12)	C5DA—N1DA—C1DA—C2DA	−54.7 (7)
C2A—C1A—C6A—C7A	−179.86 (12)	C1DA—N1DA—C3DA—C4DA	171.1 (4)
C13A—C1A—C6A—C7A	0.34 (15)	C5DA—N1DA—C3DA—C4DA	−59.7 (6)
C5A—C6A—C7A—C8A	4.1 (3)	C1DA—N1DA—C5DA—C6DA	−147.3 (6)
C1A—C6A—C7A—C8A	−178.65 (15)	C3DA—N1DA—C5DA—C6DA	86.3 (8)

Symmetry codes: (i) −*x*+1, −*y*, −*z*; (ii) −*x*+2, −*y*+1, −*z*+1.

### Hydrogen-bond geometry (Å, º)

**Table:**

*D*—H···*A*	*D*—H	H···*A*	*D*···*A*	*D*—H···*A*
O1—H1···N1*D*^iii^	0.84	1.95	2.781 (2)	171
O1—H1···N1*DA*^iii^	0.84	1.91	2.731 (2)	164
O1*A*—H1*A*···N1*B*^iv^	0.84	1.94	2.766 (2)	167
C4—H4···O1*A*^v^	0.95	2.54	3.489 (2)	175
C4*A*—H4*A*···O1^vi^	0.95	2.47	3.403 (2)	168
C9—H9···N1*C*^vii^	0.95	2.54	3.459 (2)	163
C9—H9···N1*CA*^vii^	0.95	2.60	3.519 (2)	162
C2*DA*—H2*D*6···Cl2*A*	0.98	2.90	3.819 (4)	157
C15—H15···O1	0.95	2.22	2.621 (2)	104
C15*A*—H15*A*···O1*A*	0.95	2.23	2.626 (2)	104

Symmetry codes: (iii) *x*, *y*, *z*−1; (iv) −*x*+1, −*y*+2, −*z*+1; (v) −*x*+1, −*y*+1, −*z*+1; (vi) −*x*+2, −*y*+1, −*z*; (vii) −*x*+1, −*y*+1, −*z*.

## References

[bb1] Awwadi, F. F., Willett, R. D., Peterson, K. A. & Twamley, B. (2006). *Chem. Eur. J.* **12**, 8952–8960. 10.1002/chem.200600523 16972291

[bb10] Barbour, L. J., Bourne, S. A., Caira, M. R., Nassimbeni, L. R., Weber, E., Skobridis, K. & Wierig, A. (1993). *Supramol. Chem.* **1**, 331–336.

[bb2] Bourne, S. A., Nassimbeni, L. R., Niven, M. L., Weber, E. & Wierig, A. (1994). *J. Chem. Soc. Perkin Trans. 2*, pp. 1215–1222.

[bb3] Braga, D. & Grepioni, F. (2004). In *Encyclopedia of Supramolecular Chemistry* edited by J. L. Atwood & J. W. Steed, pp. 357–363. Boca Raton: CRC Press.

[bb4] Bruker (2008). *APEX2*, *SAINT* and *SADABS*. Bruker AXS Inc., Madison, Wisconsin, USA.

[bb5] Caira, M. R., Coetzee, A., Nassimbeni, L. R., Weber, E. & Wierig, A. (1997). *J. Chem. Soc. Perkin Trans. 2*, pp. 237–242.

[bb6] Debroy, P., Lindeman, S. V. & Rathore, R. (2009). *J. Org. Chem.* **74**, 2080–2087. 10.1021/jo802579f 19191713

[bb7] Desiraju, G. R. & Steiner, T. (1999). *The Weak Hydrogen Bond in Structural Chemistry and Biology, IUCR Monographs on Crystallography*, Vol. 9. New York: Oxford University Press.

[bb8] Farrugia, L. J. (2012). *J. Appl. Cryst.* **45**, 849–854.

[bb9] Groom, C. R. & Allen, F. H. (2014). *Angew. Chem. Int. Ed.* **53**, 662–671. 10.1002/anie.201306438 24382699

[bb11] Ibragimov, B. T., Beketov, K. M., Weber, E., Seidel, J., Sumarna, O., Makhkamov, K. K. & Köhnke, K. (2001). *J. Phys. Org. Chem.* **14**, 697–703.

[bb12] James, S. L. (2004). In *Encyclopedia of Supramolecular Chemistry* edited by J. L. Atwood & J. W. Steed, pp. 1093–1099. Boca Raton: CRC Press.

[bb13] Jones, P. G. & Kuś, P. (2005). *Acta Cryst.* E**61**, o2947–o2948.

[bb14] Klien, H., Seichter, W. & Weber, E. (2013). *CrystEngComm*, **15**, 586–596.

[bb15] Klien, H., Seichter, W. & Weber, E. (2014). *Cryst. Growth Des.* **14**, 4371–4382.

[bb16] Lunazzi, L., Mazzanti, A., Minzoni, M. & Anderson, J. E. (2005). *Org. Lett.* **7**, 1291–1294. 10.1021/ol050091a 15787489

[bb17] Martinez, C. R. & Iverson, B. L. (2012). *Chem. Sci.* **3**, 2191–2201.

[bb18] Metrangolo, P., Resnati, G., Pilati, T. & Biella, S. (2008). In *Halogen Bonding, Structure and Bonding*, Vol. 126, edited by P. Metrangolo & G. Resnati, pp. 105–136. Berlin-Heidelberg: Springer.

[bb19] Mukherjee, A., Tothadi, S. & Desiraju, G. R. (2014). *Acc. Chem. Res.* **47**, 2514–2524. 10.1021/ar5001555 25134974

[bb20] Sheldrick, G. M. (2008). *Acta Cryst.* A**64**, 112–122. 10.1107/S0108767307043930 18156677

[bb21] Sheldrick, G. M. (2015). *Acta Cryst.* C**71**, 3–8.

[bb22] Skobridis, K., Theodorou, V., Alivertis, D., Seichter, W., Weber, E. & Csöregh, I. (2007). *Supramol. Chem.* **19**, 373–382.

[bb23] Toussaint, C. J. (1966). *Acta Cryst.* **21**, 1002–1003.

[bb24] Weber, E. (1996). In *Comprehensive Supramolecular Chemistry*, Vol. 6, edited by D. D. MacNicol, F. Toda & R. Bishop, pp. 535–592. Oxford: Elsevier.

[bb25] Weber, E., Nitsche, S. K., Wierig, A. & Csöregh, I. (2002). *Eur. J. Org. Chem.*, pp. 856–872.

[bb26] Weber, E., Skobridis, K., Wierig, A., Stathi, S., Nassimbeni, L. R. & Niven, M. L. (1993). *Angew. Chem. Int. Ed. Engl.* **32**, 606–608.

